# The past, present, and future of *Leishmania* genomics and transcriptomics

**DOI:** 10.1016/j.pt.2014.12.012

**Published:** 2015-03

**Authors:** Cinzia Cantacessi, Filipe Dantas-Torres, Matthew J. Nolan, Domenico Otranto

**Affiliations:** 1 Department of Veterinary Medicine, University of Cambridge, Cambridge, UK; 2 Departamento de Imunologia, Centro de Pesquisas Aggeu Magalhães, Fiocruz-PE, Brazil; 3 Dipartimento di Medicina Veterinaria, Università degli Studi di Bari, Bari, Italy; 4 Royal Veterinary College, University of London, North Mymms, UK

**Keywords:** leishmaniases, *Leishmania infantum*, high-throughput sequencing, genome, transcriptome, bioinformatics, sand fly, metazoonosis, host-parasite interactions, One Health

## Abstract

It has been nearly 10 years since the completion of the first entire genome sequence of a *Leishmania* parasite. Genomic and transcriptomic analyses have advanced our understanding of the biology of *Leishmania*, and shed new light on the complex interactions occurring within the parasite–host–vector triangle. Here, we review these advances and examine potential avenues for translation of these discoveries into treatment and control programs. In addition, we argue for a strong need to explore how disease in dogs relates to that in humans, and how an improved understanding in line with the ‘One Health’ concept may open new avenues for the control of these devastating diseases.

## Highlights

• Genomic and transcriptomic technologies are changing *Leishmania* research.
• Research into new control strategies provides insights into the vector–parasite–host triangle.
• Pathways of infection and disease in human and canine leishmaniases must be clarified.

## Burden of leishmaniasis and the need for a ‘One Health’ initiative

Leishmaniases are a group of diseases caused by digenetic protozoa of the genus *Leishmania*, which are transmitted by phlebotominae sand flies (Table 1). Based on recent estimates, up to 0.4 million and 1.2 million cases of visceral (VL) and cutaneous leishmaniasis (CL), respectively, occur each year in 98 countries and three territories where these diseases are endemic [1]. Despite their widespread distribution, over 90% of global VL cases occur in only six countries (India, Bangladesh, Sudan, South Sudan, Ethiopia, and Brazil), while most cases (70–75%) of CL occur in ten countries (Afghanistan, Algeria, Colombia, Brazil, Iran, Syria, Ethiopia, North Sudan, Costa Rica, and Peru) [1]. In most cases, leishmaniases are zoonoses, affecting the poor in rural and natural areas, where a plethora of domestic and wild reservoir hosts and sand fly vectors maintain the infection [2]. For instance, 13 out of the 21 human-infective *Leishmania* have also been reported in domestic dogs, the latter having a major role in maintaining and transmitting the infection to other receptive hosts via the sand fly vectors [3] (Table 1). In accordance with the concept of ‘One Health’, defined as ‘a movement to forge co-equal, all inclusive collaborations between physicians, […], veterinarians and other scientific-health and environmentally related disciplines […] to improve and defend the health and well-being of all species’ (http://www.onehealthinitiative.com), successful control strategies against human leishmaniases must include preventative measures focussed on the human and animal hosts and arthropod vectors, as well as on the environments where the latter perpetuate [3]. To achieve these goals, a thorough understanding of the host–pathogen–vector triangle, and particularly of their intimate interactions at the molecular level, is imperative. Recent advances in -omics technologies, including genomics and transcriptomics, together with the considerable decrease in the cost of these techniques, provide exciting opportunities to reveal details of the intimate relations between *Leishmania* parasites, human and animal hosts, and sand fly vectors. In this review, we provide an overview of a range of milestone studies that have used genomics and transcriptomics techniques to improve current understanding of the biology of *Leishmania*, as well as of the molecular interactions between this parasite and its vertebrate and arthropod hosts. In addition, given the intimate relations between human and canine leishmaniases in endemic areas, and in line with the ‘One Health’ movement, we argue that current and future efforts should be directed towards integrating -omics technologies (i.e., genomics, transcriptomics, proteomics, metabolomics, and interactomics) to achieve a better understanding of the similarities and differences between human and canine infections, with the ultimate aim of developing new diagnostics, and treatment and control strategies against this devastating group of diseases.

## The fight against leishmaniasis: how can -omics help?

The control of leishmaniases generally relies on the early diagnosis and treatment of human cases, vector control, and, in some cases, management of reservoir hosts (i.e., treatment and/or elimination) [3]. However, the control of leishmaniases, as with any vector-borne disease, is not trivial due to challenges relating to intervention programs, mostly in developing countries, where the burden of disease is heavier (due to a combination of factors including, but not limited to, a lack of political will, of human resources, and of infrastructure). In addition, our limited knowledge of the host–pathogen–vector triangle, particularly of their intimate interactions at the molecular level, impairs the development of more affordable and effective control tools, such as antivector vaccines and more effective chemotherapeutics.

-Omics technologies are increasingly being applied to investigations of determinants of disease phenotype [4], mode of action of current drugs [5], and parasite biology [6]. These studies have improved our understanding of the pathogenesis of disease in humans and possible mechanisms of resistance to antileishmanial drugs. Without a doubt, -omics approaches are likely to reveal details of the intimate relations between hosts, parasites, and vectors. This refined knowledge will foster the development of new control tools (e.g., antivector vaccines) that could assist the fight against leishmaniases. The determination of the whole genome sequences of a range of *Leishmania* parasites causing both VL and CL represents the first step towards these goals, providing the scientific community with a solid infrastructure for postgenomic investigations of the parasite biology, pathogenicity, and mastery mechanisms of manipulation of both insect and vertebrate hosts.

## The *Leishmania* genomes: a ‘toolbox’ to understand host–parasite interactions

Efforts to determine the whole genome sequence of key *Leishmania* species infecting humans were consolidated in 1994 in Rio de Janeiro (Brazil), with the establishment of the *Leishmania* Genome Network (LGN) initiative. Not only did this network represent the researchers’ first move to expand existing knowledge of the fundamental molecular biology of this parasite, with a view towards promoting the discovery of novel treatment and control strategies, but it also saw the support of the FIOCRUZ and UNICEF/UNDP/World Bank/WHO Special Programme for Research and Training in Tropical Diseases [7]. In 2005, these efforts proved successful, with the publication of the first complete genome sequence of *Leishmania major* (causing CL) [8], soon followed by those of *Leishmania infantum* (causing VL) and *Leishmania braziliensis* (causing mucocutaneous leishmaniasis; MCL) [9]. In recent years, the advent of high-throughput sequencing technologies (Box 1) has assisted relentless progress in the genomics of human leishmaniases, with the completion of the whole genome sequences of *Leishmania mexicana* (CL; [10]), *Leishmania donovani* (VL; [11]) and *Leishmania amazonensis* (CL; [12]) (Box 1). The availability of these genome sequences has provided unprecedented opportunities to perform detailed comparative analyses of *Leishmania* species associated with different human diseases at a scale previously unimaginable [9,12].

**Figure 1:**
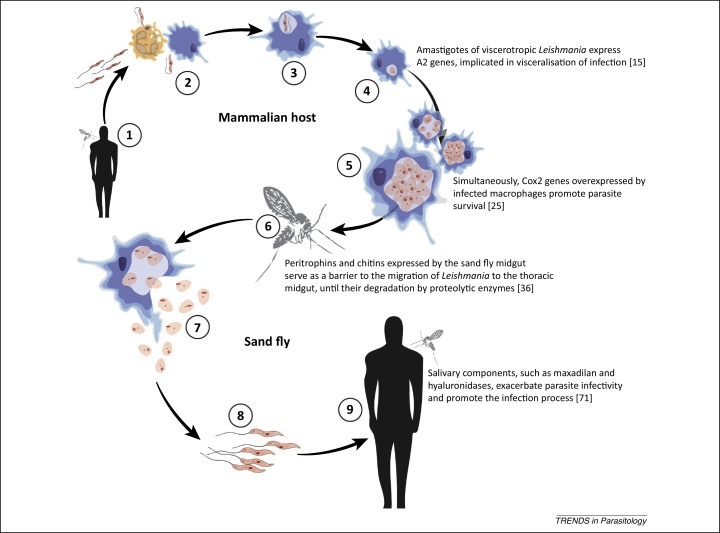
Life cycle of *Leishmania* spp. and examples of molecules putatively involved in parasite infectivity and visceralisation of infection. Phlebotominae sand flies release *Leishmania* infective stages (i.e., metacyclic promastigotes) to the mammalian hosts during blood feeding (1); the parasites invade macrophages and granulocytes (2 and 3) and develop to amastigotes inside the phagolysosome (4); the amastigote stages replicate within the phagolysosome by simple division (5); then, amastigote-containing macrophages are ingested by susceptible sand flies during the blood meal (6); the parasites are released from the infected macrophages within the sand fly midgut (7), where they transform into procyclic promastigotes and divide. Then, the parasites migrate towards the stomodeal valve (anterior midgut) and transform into different promastigote subtypes that ultimately form metacyclic promastigotes (8). These infective stages are then released into a new mammalian host during a subsequent blood meal (9) [15,25,36,71]. Abbreviation: Cox2, prostaglandin-endoperoxide synthase 2.

The genomes of *Leishmania* vary from 29 Mb (*L. amazonensis*; [12]) to 33 Mb in size (*L. major*, *L. infantum* and *L. braziliensis*; [9]) and are organised into a variable number of chromosomes (i.e., 34 in *L. amazonensis* and *L. mexicana*, 35 in *L. brasiliensis*, and 36 in *L. major*, *L. donovani*, and *L. infantum*) [12]. Despite the striking variability in pathogenicity and tissue tropism of different *Leishmania* species, their genomes are remarkably similar, displaying a high degree of conservation in gene content and architecture (synteny) [9,12]. The genomes of *Leishmania* spp. are characterised by a high gene density, the presence of long arrays of polycistronic gene clusters, and the almost complete absence of introns [7]. However, careful examination of protein-coding genes in *Leishmania* allowed the identification of a relatively small number of species-specific genes, the majority of which encode predicted proteins of unknown function [10]. Only a few of these genes could be associated to specific tissue tropism. For instance, *LinJ*.28.0340, a gene specific to *L. infantum* and occurring as a pseudogene in *L. major*, *L. braziliensis*, and *L. mexicana* [10], has been implicated in the ability of the latter to spread and survive in visceral organs of the vertebrate hosts [13]. Indeed, when a *L. donovani* gene orthologue of *LinJ*.28.0340 was introduced into transgenic *L. major*, the latter displayed a significantly increased capacity to survive in visceral organs of BALB/C mice [13]. Conversely, the spleens and livers of mice infected with the *LinJ*.28.0340/*L. donovani* null mutant were characterised by significantly reduced parasite burdens compared with those infected with the wild type *L. donovani* counterpart, thus providing solid evidence for a role of this gene in the visceralisation of the infection [13]. Among other genes thought to have key roles in the ability of species within the *L. donovani* complex to colonize visceral organs, those belonging to the A2 gene family are also present as pseudogenes in *L. major* [14]. These genes were first identified in *L. donovani* and shown to be exclusively expressed by the amastigote stage (*cf*. [14]) (Figure 1); subsequently, these genes were demonstrated to be essential for the survival of *L. donovani* in visceral organs, while transgenic *L. major* expressing A2 genes displayed increased survival in the spleens of infected mice [14]. Despite the evidence for a role of A2 genes in the pathogenesis of VL, molecules encoding A2 proteins have also been identified in *Leishmania* species responsible for CL, such as *L. amazonensis* and *L. mexicana* [10,12]. While the presence of these proteins in *Leishmania* parasites with skin tropism has been attributed to functional divergence between Old World and New World species [14], their role in the pathogenesis of CL is yet to be ascertained. Interestingly, a recent comparative analysis of the genomes and transcriptomes of two phenotypically distinct substrains of *L. donovani* (i.e., one causing VL and one responsible for a large number of cases of CL in Sri Lanka) revealed an increased copy number of A2 genes in *L. donovani* causing VL, which was also associated with significant upregulation in A2 mRNA transcription and protein expression in strains causing VL [15]. In the same study, Zhang and colleagues [15] identified the presence of several nonsynonymous SNPs in genes from the *L. donovani* CL strain. Among these was a molecule encoding a ras-like small GTPase-RagC protein; insertion of the corresponding orthologous gene from the *L. donovani* VL isolate into the CL counterpart resulted in a significant increase in parasite burdens in the spleen of infected mice [15]. These data provided evidence of the impact of SNPs on gene function and phenotype, thus refining current understanding of their potential impact on the pathogenicity of different strains of *Leishmania*.

While comparative analyses of the whole genome sequences of *Leishmania* species causing CL and VL represent a solid basis for in-depth investigations of the intimate mechanisms of host–parasite interactions that result in different courses of infection, studies of the regulation of parasite gene expression throughout its life cycle in both the vertebrate hosts and the sand fly vectors are likely to contribute to a better understanding of the pathogenesis of disease. Clearly, the availability of an array of genomes, together with an explosion in microarray and high-throughput transcriptomic sequencing technologies, have facilitated such studies (e.g., [16–19]). However, the same organisation into polycistronic transcription units that makes the genomes of CL- and VL-causing *Leishmania* so strikingly similar [7] has been deemed responsible for the lack of extensive gene expression regulation at the transcriptional level [20]. Indeed, most *Leishmania* genes have been shown to be constitutively expressed throughout the transition from promastigote to amastigote stage [21], with post-transcriptional events, including mechanisms that control the abundance of mRNAs, translation rates and post-translational protein stability, hypothesised to have key roles in the regulation of protein abundance [21]. However, the marked variation in chromosome and gene copy numbers among strains of *L. infantum*, *L. mexicana*, *L. braziliensis*, and *L. major* unveiled, for the first time, a degree of aneuploidy in the genomes of these parasites [10]. Accordingly, unstable ploidy among strains of *L. infantum*, as well as variable chromosomal contents among cells, revealed that the *Leishmania* genome is characterised by ‘mosaic aneuploidy’ [11,22]. Therefore, ‘genome plasticity’ and ‘gene dosage’, rather than differential expression of single genes and gene products, are increasingly being considered as two of the keys to the different tissue tropism of *Leishmania* spp. [22].

## Transcriptomics unveils *Leishmania*-mediated regulation of host gene expression

Several transcriptomic studies have investigated *Leishmania*-induced regulation of gene expression in infected tissues with the aim to link such responses to disease outcome. As an example, for VL-causing *Leishmania*, Beattie and colleagues [23] used whole-genome array technologies to compare the gene expression profiles of liver-resident macrophages (Kupffer cells) from mice infected by *L. donovani* to those of uninfected macrophages exposed to inflammatory stimuli. The authors showed significant upregulation of genes within the retinoid X receptor α pathway (i.e., *Rxra*), linked to lipid metabolism, in uninfected macrophages exposed to inflammation compared with the infected counterpart [23]; pharmacological perturbation of the activity of this pathway in Kupffer cells resulted in an increased resistance of these cells to *Leishmania* infection, which led to speculation that either this pathway has a role in the usage of lipids and cholesterol by the parasite, or that *Leishmania* lipids regulate the activation of innate immune responses that follow the infection [23]. For CL-causing *Leishmania*, Maretti-Mira and colleagues [24] utilised high-throughput RNA-Seq technologies to characterise and compare the transcriptomes of tissue fragments obtained from human subjects with CL and MCL caused by *L. braziliensis* [24]. The outcomes from this study highlighted significant upregulation of genes involved in biological pathways linked to the recruitment and activation of immune cells (including lymphocytes, granulocytes, natural killer cells, and antigen-presenting cells) and to regulation of inflammatory responses in tissues from subjects with CL [24]. This suggested that the inability of the host to mount effective immune responses against the parasite at the site of cutaneous infection is linked to the progression of disease [24]. In an effort to characterise differences in macrophage gene expression that might contribute to the ability of different *Leishmania* spp. to cause localised (CL) or systemic infections (VL), Gregory and colleagues [25] used a DNA microarray approach to perform comparative analyses of the transcriptomes of murine macrophages infected by *L. major* and *L. donovani*. Interestingly, both parasites induced a similar differential regulation of relatively small numbers of macrophage genes, with most of these genes unsurprisingly linked to the development of immune responses [25]. The only noticeable difference in gene expression profiling between *L. major*- and *L. donovani*-infected macrophages was a remarkable increase in levels of transcription of mRNAs encoding prostaglandin-endoperoxide synthase (Cox2) in the latter, which led to speculation that this pathway is involved in the pathogenesis of VL [25] (Figure 1).

Clearly, the availability of high-throughput transcriptomic technologies has resulted in rapid expansion of the already substantial plethora of knowledge of the molecular interactions occurring between *Leishmania* and the human host; nevertheless, significant variation in host responses to infection has been described in several studies (*cf*. [26]), although a review of this variation is beyond the scope of the present article. However, these technologies have also enabled progress towards the exploration of the molecular relationships between the parasite and the sand fly vector and the patterns of *Leishmania* development into its infective, nondividing metacyclic form [27].

## Transcriptomics in *Leishmania*–sand fly interactions

Transmission of *Leishmania* from an infected to a susceptible host requires development of the parasites in the midgut of a competent sand fly vector. Macrophages containing *Leishmania* amastigotes are ingested by sand fly vectors via a blood meal and, once released in the insect midgut, develop through several developmental stages into infective, metacyclic promastigotes [26] (Figure 1). The reproductive mode of *Leishmania* parasites has traditionally been considered clonal, based on strong linkage disequilibrium (*cf*. [28]); however, several studies have provided solid evidence of the occurrence of genetic exchange between species and/or strains of *Leishmania* (i.e., *L. major* and *L. infantum*) during growth and development in the sand fly vector, with successful transmission of the hybrid progeny to a susceptible vertebrate host [28–32]. The range of vertebrate and invertebrate host species that *Leishmania* can infect, as well as the multiple forms of disease that it causes, have been partly attributed to the ability of this parasite to undergo genetic exchange in the sand fly vector (*cf*. [29]). Clearly, the molecular interactions that occur at the parasite–sand fly interface are key processes that determine the successful development and transmission of *Leishmania*; therefore, a detailed understanding of these mechanisms has become a priority. Previous studies had used Sanger sequencing of cDNA libraries from the midgut of sand fly vectors of both CL- and VL-causing *Leishmania* (i.e., *Phlebotomus papatasi*, vector of *L. major* and *Lutzomyia longipalpis*, vector of *L. infantum*; [33,34]) to identify molecules putatively involved in the development of the parasites in their insect vectors. While sand fly infections by *L. major* and *L. infantum* were consistently associated with downregulation of molecules encoding microvilli-like proteins and chymotrypsin and upregulation of trypsin-encoding transcripts, the transcription profiles of peritrophin-like molecules were inconsistent between *P. papatasi* and *Lu. longipalpis* [33,34]. Peritrophins are the protein component of the peritrophic matrix (PM), an extracellular chitin-containing structure that encapsulates the blood meal following its ingestion by the sand fly [35]. The formation of the PM (immediately following the blood meal) has long been considered advantageous for *Leishmania*, because the parasites are thought to be protected from the action of the sand fly proteolytic enzymes during the vulnerable time of development to promastigotes [35]. Several key investigations have contributed to further understanding of the relations between *Leishmania* promastigotes and the sand fly PM (e.g., [36]). In particular, while previous studies hypothesised a role of *Leishmania* chitinases in the disintegration of the sand fly PM (*cf*. [36]), current evidence supports the theory that the breakdown of the PM is independent from the activity of *Leishmania* enzymes and that parasite promastigotes escape the PM by migrating through a posterior opening that forms irrespective of the infection status of the sand fly [36]. In the same study, Sadlova and Volf [36] showed that the anterior plug of the PM serves as a ‘barrier’ for parasite migration to the thoracic midgut, until its degradation from sand fly proteolytic enzymes is complete [36]. The elucidation of patterns of sand fly gene expression during the disintegration of the PM in the presence (or not) of *Leishmania* parasites, and during migration of the latter from the abdominal to the thoracic midgut, may help to either confirm or confute this point.

Together with studies of the midgut of sand flies, other investigations used transcriptomic technologies to shed light on the molecular mechanisms that govern the development of *Leishmania* parasites into their infective metacyclic stage [37]. While little information is available on sand fly molecular pathways acting as trigger of *Leishmania* metacyclogenesis, recent studies highlighted the role of key genes and gene products in the differentiation of promastigote stages into metacyclic forms in the sand fly vector. Among these molecules, a hydrophilic acylated surface protein (HASPB) and a small hydrophilic endoplasmic reticulum (ER)-associated protein (SHERP) showed increased expression in the metacyclic stages [38]; in addition, creation of HASPB and SHERP null mutants in *L. major* resulted in the accumulation of non-infective parasite stages in the midgut of the sand fly vector, thus providing evidence for the essentiality of these molecules for parasite development [38]. Investigations of patterns of gene transcription during *Leishmania* metacyclogenesis *in vitro* have led to the identification of genes and gene products potentially related to parasite infectivity (e.g. [37,39–41]). For instance, recent functional studies of essential molecules in *L. major* metacyclic promastigotes highlighted major roles of mitogen-activated protein kinases (i.e., MAPK4) and metallopeptidases of the M24A family in the establishment of intracellular macrophage infections [40] and proliferation in infected cells [41]. These data provided a solid basis for the exploration of the role of these molecules as novel targets for intervention strategies.

In recent years, the search for new and effective preventative measures against *Leishmania* transmission has also involved the characterisation of key components of the saliva of the sand fly vectors (e.g., [27,42–46]). The interest of the scientific community in salivary gland transcriptomes (‘sialotranscriptomes’) is mainly derived from knowledge that selected saliva proteins have crucial roles in facilitating the successful establishment of *Leishmania* parasites in vertebrate hosts, including the regulation of the immune response at the site of bite [27,46,47]. Therefore, sialotranscriptomes of several competent sand fly vector species are now available (e.g., [43–45,48–51]), which, in some cases, have led to the selection of key sand fly molecules that are involved in the blood-feeding process and that may assist the immunoevasive strategies of *Leishmania* [47,52] (Box 2). For instance, a potent vasodilator (maxadilan) abundantly detected in the saliva of *Lu. longipalpis* [53] has not been identified in transcriptomic data sets from the salivary glands of *Lutzomyia ayacuchensis* [44]. Similarly, a maxadilan homologue identified in *Lutzomyia intermedia* showed only 34% identity to maxadilan from *Lu. longipalpis* [50]. It is worth noting that both *Lu. intermedia* and *Lu. ayacuchensis* are vectors of dermotropic *Leishmania* species, whereas *Lu. longipalpis*, whose saliva contains large amounts of maxadilan, is the main vector of the viscerotropic *L. infantum* in the New World [54] (Figure 1). While these observations suggested a role of maxadilan in visceralisation of *L. infantum* infection [55], the absence of maxadilan homologues from the saliva of sand fly vectors of VL in the Old World raises questions about the role/s of other salivary components in disease progression. Indeed, other enzymes, such as hyaluronidases and apyrases, have been identified using transcriptomic and proteomic technologies from several sand fly vectors of VL in both the Old and New Worlds [45]. These enzymes have been shown to positively contribute to the spread of *Leishmania* parasites by promoting the enlargement of the feeding lesion and the diffusion of other salivary active compounds (hyaluronidases) and preventing haemostasis (apyrases) [45].

Besides containing components essential to the infection process, the saliva of sand flies contains molecules that can elicit specific immune responses that are indicative of host exposure to sand fly bites (e.g., [56,57]). In particular, three proteins from the saliva of *P. perniciosus* (i.e., two yellow proteins and an apyrase), expressed in recombinant form, were shown to be useful in determining the intensity of exposure to sand fly bites in experimentally bitten mice and dogs [57]. While cross-reactivity between anti-*P. perniciosus* antibodies and those from closely related sand fly species was not assessed [57], the authors hypothesised that this may occur. Both yellow proteins and apyrases have been detected in the saliva of a range of sand fly species. However, subtle differences in sequence may result in varying immunogenic properties; future investigations using transcriptomic and proteomic technologies may assist elucidating this point via, for instance, the generation of whole transcript and/or protein data sets from sand fly vector species, with the ultimate aim of identifying suitable targets for the development of commercial diagnostic tools to assess the risk of human and canine transmission in both endemic and nonendemic areas, and the evaluation of the effectiveness of antivector campaigns [56]; this improved knowledge could also aid current efforts aimed at developing recombinant vaccines containing immunogenic components from both the parasite and the sand fly vectors. In addition, thus far, no data are available on the effects of *Leishmania* infections on the global transcriptional profiles of sand fly vectors. Future studies could, for instance, utilise RNA-Seq technologies to investigate differences in gene expression profiling of *Leishmania*-infected and uninfected sand flies. Exploring and identifying molecular pathways involved in the parasite–vector–host interactions may lead to the identification of new molecular pathways implicated in the infection process, which would be instrumental for refining current control strategies against sand flies. Undoubtedly, some challenges exist in performing large-scale transcriptomic studies of species for which a reference genome is unavailable; among these challenges, the *de novo* assembly of full-length transcripts in absence of reference sequences is one of the most significant [58]. Nevertheless, other resources, such as the genomes and transcriptomes of selected mosquito species [59] that are phylogenetically related to sand fly vectors of *Leishmania* [60], could be exploited for the accurate reconstruction of (at least) a proportion of full-length sand fly transcripts, thus reducing overall project costs and limiting potential biases introduced by *de novo* assembly.

## Concluding remarks and research needs

Over the past decade, advances in genomics and transcriptomics technologies have contributed to considerably enhance our knowledge of the set of molecular interactions that occur within the host–parasite–vector triangle. However, some gaps still exist in our understanding of the similarities and/or differences between human leishmaniases and the disease in animal reservoir hosts. Dogs, for instance, represent the most important host reservoir for *L. infantum* (causing VL) [3,61]. Therefore, differences and similarities between human and canine infections should be comprehensively analysed. However, most studies of *Leishmania* immunobiology and genetics, as well as of host–parasite interactions, utilise murine models of infection as ‘mirrors’ of human disease [61]. Given that transmission of key *Leishmania* species (e.g., *L. infantum*) to humans strictly relies on the circulation of the parasite among canine populations, elucidating whether dog leishmaniasis serves as a model for human infections should become a priority. This could provide avenues for studies aimed, for instance, at evaluating the ‘translatability’ of novel treatment and vaccine strategies from humans to dogs and vice versa. The availability of *in vivo* canine models of leishmaniasis [62], together with advances in genomics and/or transcriptomics, proteomics, and metabolomics technologies, may assist this quest. For instance, RNA-Seq and high-throughput proteomics platforms provide a golden opportunity to monitor changes in host gene transcription and protein expression throughout the course of canine and human infections, thus enabling one to draw parallels between them. Similarly, large-scale analyses of metabolites produced during the course of infection, both by the parasite and the vertebrate host, may represent a gold mine for the identification of novel diagnostic biomarkers, as well as of potential new *Leishmania* ‘Achilles’ heels’ that could assist current programs aimed at breaking the transmission cycle of human and canine leishmaniases.

## Figures and Tables

**Table 1:** Principal causative agents of human leishmaniases

*Leishmania* species	Principal tropism^a^	Geographical distribution^b^	Notes on the infection in dogs^c^
*Leishmania aethiopica*	C	Old World: Ethiopia, Kenya	
*Leishmania amazonensis*	C	New World: Argentina, Bolivia, Brazil, Colombia, Ecuador, French Guiana, Peru, Suriname, Venezuela	VL cases in Brazil
*Leishmania archibaldi*^d^	V	Old World: Ethiopia, Kenya, Lebanon, Sudan	VL cases in Sudan
*Leishmania braziliensis*	C, MC	New World: Argentina, Belize, Bolivia, Brazil, Colombia, Costa Rica, Ecuador, Guatemala, French Guiana, Honduras, Mexico, Nicaragua, Panama, Paraguay, Peru, Venezuela	CL cases in Argentina, Bolivia, Brazil Colombia, Peru, and Venezuela
*Leishmania colombiensis*	C	New World: Colombia, Panama, Venezuela	VL in a dog in Venezuela
*Leishmania donovani*	V	Old World: Bangladesh, Bhutan, China, Cyprus, Djibouti, Ethiopia, India, Iraq, Israel, Kenya, Nepal, Saudi Arabia, Somalia, Sri Lanka, Sudan, Ukraine, Uganda, Yemen	Dogs are commonly infected in some countries (e.g., Sudan), but their role as reservoirs is unknown
*Leishmania garnhami*^d^	C	New World: Costa Rica, Venezuela	
*Leishmania guyanensis*	C	New World: Argentina, Bolivia, Brazil, Colombia, Ecuador, French Guiana, Guyana, Peru, Suriname, Venezuela	CL cases in Colombia
*Leishmania infantum*	V, C	Old World: Afghanistan, Albania, Algeria, Armenia, Azerbaijan, Bosnia and Herzegovina, Bulgaria, Central African Republic, China, Cyprus, Croatia, Egypt, France, Gambia, Georgia, Greece, Iraq, Iran, Israel, Italy, Libyan Arab Jamahiriya, Jordan, Kazakhstan, Kirgizstan, Lebanon, Macedonia, Malta, Morocco, Mauritania, Monaco, Montenegro, Oman, Pakistan, Palestine, Portugal, Syria, Romania, Senegal, Saudi Arabia, Slovenia, Spain, Sudan, Tunisia, Turkmenistan, Turkey, Ukraine, Uzbekistan, Yemen. NEW WORLD: Argentina, Bolivia, Brazil, Colombia, Costa Rica, El Salvador, Guatemala, Honduras, Mexico, Nicaragua, Paraguay, Venezuela	VL cases usually found in areas where human cases are reported. Autochthonous cases reported in dogs in the USA (no human cases reported so far)
*Leishmania killicki*^d^	C	Old World: Algeria, Libyan Arab Jamahiriya, Tunisia	
*Leishmania lainsoni*	C	New World: Bolivia, Brazil, French Guiana, Peru, Suriname	
*Leishmania lindenbergi*	C	New World: Brazil	
*Leishmania major*	C	Old World: Afghanistan, Algeria, Azerbaijan, Burkina Faso, Cameron, Chad, Egypt, Ethiopia, Georgia, Ghana, Guinea, Guinea-Bissau, India, Iraq, Israel, Libyan Arab Jamahiriya, Jordan, Kazakhstan, Kenya, Kuwait, Mali, Morocco, Mauritania, Mongolia, Niger, Nigeria, Oman, Pakistan, Palestine, Saudi Arabia, Syria, Iran, Senegal, Sudan, Tunisia, Turkmenistan, Uzbekistan, Yemen	CL in Egypt and Saudi Arabia
*Leishmania mexicana*	C	New World: Belize, Colombia, Costa Rica, Ecuador, Guatemala, Mexico, United States	CL in Ecuador and USA
*Leishmania naiffi*	C	New World: Brazil, French Guiana,	
*Leishmania panamensis*	C, MC	New World: Colombia, Costa Rica, Ecuador, Guatemala, Honduras, Nicaragua, Panama	CL in Ecuador and Colombia
*Leishmania peruviana*	C	New World: Peru	CL in Peru
*Leishmania pifanoi*^d^	C	New World: Venezuela	CL in Ecuador
*Leishmania shawi*	C	New World: Brazil	
*Leishmania tropica*	C	Old World: Afghanistan, Azerbaijan, Egypt, Ethiopia, Greece, India, Iraq, Israel, Iran, Jordan, Kenya, Morocco, Namibia, Pakistan, Palestine, Saudi Arabia, Syria, Turkmenistan, Turkey, Uzbekistan, Yemen	CL cases in India, Iran, Israel, Morocco, and Syria
*Leishmania venezuelensis*	C	New World: Venezuela	

a Abbreviations: C, dermotropic; MC, mucotropic; V, viscerotropic.

b Based on [63,64].

c Based on [54,65,66]. In addition, *Leishmania arabica* has been reported in dogs in Saudi Arabia [67]. Moreover, other *Leishmania* species (e.g., *Leishmania equatorensis* and *Leishmania utingensis*) [68,69] have been described from wildlife and/or sand flies, but have not yet been detected in humans or dogs.

d Species status is under discussion [63,70].

## Box 1 DNA sequencing technologies and Leishmania genomics: a decade of progress

In 2005, the *Leishmania major* genome was published in the journal *Science* [8]. The successful completion of the first whole genome sequence of a *Leishmania* species resulted from a collaboration between the Wellcome Trust Sanger Institute (UK), the WTSI-coordinated EULEISH consortium, and the Seattle Biomedical Research Institute (USA) [8]. The sequencing strategy utilised a combination of high- and low-coverage large insert clone sequencing and a whole chromosome shotgun approach, preceded by purification of single or co-migrating chromosomes using pulse-field gel electrophoresis (PFGE) [8]. In 2007, the genomes of two additional *Leishmania* species, those of *Leishmania infantum* and *Leishmania braziliensis*, were completed by a multi-institutional network of scientists led by the WTSI [9]; to generate an approximate sixfold coverage of the complete genome sequences of these two species, researchers utilised whole-genome shotgun sequencing of plasmid clones containing genome fragments of variable length (up to 4 kb). Four years later, a similar approach was used to sequence the genome of *Leishmania mexicana* [10]. In the same year, the reference genome of *Leishmania donovani* was the first to be completed using a next-generation sequencing strategy [11]; in particular, using a pyrosequencing approach (454 Roche), Downing and colleagues generated a total of 1.29 million single-end and 3.58 million paired-end reads [with an average length of 167 base pairs (bp)], 96% of which were assembled into an initial set of reference contigs and scaffolds [11]; subsequently, 17 clinical isolates of *L. donovani* obtained from Nepalese and Indian patients with VL (including the isolate from which the reference genome sequence was obtained), were sequenced using high-throughput Illumina sequencing, generating a total of 41 Gb of sequence data [11]. Then, the 76-bp paired-end reads from each isolate were mapped to the reference genome sequence for SNP analysis, which resulted in new insights into *Leishmania* population genetics and mechanisms of emerging drug resistance [11]. The most recent *Leishmania* genome sequence (i.e., that of *Leishmania amazonensis* [12]) was generated using a combination of 454 and Illumina sequencing. The approximately 179 000 454 reads corresponded to a twofold coverage of the *L. amazonensis* genome and were assembled into 27 856 contigs that, together with the 4411 scaffolds derived from the assembly of approximately 37 million 76-bp paired-end Illumina reads, resulted in the final assembly of 2627 scaffolds [12].

## Box 2 Sand-fly salivary gland secreted proteins and Leishmania: examples from a successful partnership

The salivary glands of sand fly vectors secrete several proteins known to facilitate the process of host invasion by *Leishmania* [27]. Among these proteins, maxadilan, a known vasodilator, was first described in *Lutzomyia longipalpis* and shown to exacerbate the infectivity of *Leishmania major* via the inhibition of lymphocyte proliferation [47]. Since this first report, a range of other molecules has been described from the salivary glands of an array of sand fly species that contribute to the successful establishment of *Leishmania* infections. Among these molecules, protein homologues of salivary hyaluronidases secreted by *Phlebotomus papatasi* were demonstrated to have a role in the severity of skin lesions caused by *L. major* [71]. While the mechanisms that result into this outcome are yet to be elucidated, Volfova and colleagues [71] hypothesised that the neutrophils recruited through the enzymatic activity of sand fly hyaluronidases (via the presence of hyaluronan fragments) may be exploited by *Leishmania* as ‘Trojan horses’, thus facilitating macrophage infection [71]. Interestingly, a secreted endonuclease identified from the sialotranscriptome of *Lu. longipalpis* has been recently implicated in the ability of *Leishmania* to evade killing by neutrophils [47]. This protein was shown to interfere with the microbicidal activity of neutrophils recruited at the site of a sand fly bite by disintegrating the neutrophil extracellular traps (NETs) that ensnare *Leishmania* parasites, thus significantly contributing to the immunoevasive strategies of the parasite and enhancing its infectivity [47].
